# Fs laser written volume Raman–Nath grating for integrated spectrometer on smartphone

**DOI:** 10.1038/s41598-023-40909-9

**Published:** 2023-08-22

**Authors:** Jean-Sébastien Boisvert, Sébastien Loranger, Raman Kashyap

**Affiliations:** 1 Department of Engineering Physics, Ecole Polytechnique Montréal, 2900 Édouard-Montpetit, Montreal, QC H3T 1J4 Canada; 2 Department of Electrical Engineering, Poly-Grames, Ecole Polytechnique Montréal, 2900 Édouard-Montpetit, Montreal, QC H3T 1J4 Canada

**Keywords:** Integrated optics, Optical sensors, Laser material processing

## Abstract

In this work we demonstrate the integration of a spectrometer directly into smartphone screen by femtosecond laser inscription of a weak Raman–Nath volume grating either into the Corning Gorilla glass screen layer or in the tempered aluminosilicate glass protector screen placed in front of the phone camera. Outside the thermal accumulation regime, a new writing regime yielding positive refractive index change was found for both glasses which is fluence dependent. The upper-bound threshold for this thermal-accumulation-less writing regime was found for both glasses and were, respectively at a repetition rate less than 150 kHz and 101 kHz for fluence of 8.7 × 10^6^ J/m^2^ and 1.4 × 10^7^ J/m^2^. A weak volume Raman–Nath grating of dimension 0.5 by 3 mm and 3 μm pitch was placed in front of a Samsung Galaxy S21 FE cellphone to record the spectrum using the 2nd diffraction order. This spectrometer covers the visible band from 401 to 700 nm with a 0.4 nm/pixel detector resolution and 3 nm optical resolution. It was used to determine the concentration detection limit of Rhodamine 6G in water which was found to be 0.5 mg/L. This proof of concept paves the way to in-the-field absorption spectroscopy for quick information gathering.

## Introduction

Since their introduction in 1993^1^, smartphones have become devices that are widely used and integrated into our daily life all around the world. This integrated platform has evolved over the years by the increase of its computing power capabilities and the addition of new sensors and features. It has already replaced common commodity items such as the video or photographic cameras, alarm clocks, watches, Global Positioning Systems (GPS), calendars, the calculator, flash lamps, just to name a few becoming as powerful as a small computer with access to the web. The recent Covid pandemic has highlight the potential of this tool to rapidly implement and distribute applications to a vast population in record time.

Photonics can be an interesting avenue to increase the capabilities and thus the potential of these devices. Manufacturers already have integrated new photonics sensors such as Lidars for augmented reality applications or pulse oximeter to acquire on field blood oxygen level and cardiac frequency to some recent model of smartphones. Simultaneously, many research groups are actively working to create new functionalities on this device using the already on-board sensors or developing new ones. Microscopy systems using smartphone cameras coupled with an algorithm have been demonstrated to count white or red blood cell^2^ for blood sample analysis as well as detection of parasites^3^, bacteria^4,5^ and viruses^6^. Blood sugar level can be detected by evaluating the ratio of the blue and green spectral components at the RGB camera^7^. Turbidity level of water using Mie diffusion can be also measured as shown in^8^. An optical breathalyzer based on the difference in evaporation rate with alcohol content of the fog generated in breath, was as well demonstrated^9^. Spectroscopy systems capable of measuring the water pH level have been also demonstrated with a resolution of 0.305 nm/pixel^10^. Detection of water contaminant like copper, chrome, fluor, lead, mercury or pesticides^11^ was also investigated. Plasmonic resonance systems can be coupled with spectroscopy to detect agents that are transparent to the optical bandwidth of the camera and offer low concentration detection level of analyte in water (100 picogram/mL) of staphylococcal enterotoxin B as reported in^12^.

However, these new functionalities often need additions of component as an add-on which consumes space. The space limitation problem is of concern in conditions when optimized devices are required. To tackle this issue, the idea of using the 750 μm thick protecting layers made of Corning Gorilla glass in front of the screen to inscribe photonics devices was proposed by Lapointe et al. in^13^. With the help of femtosecond (fs) laser writing at 1030 nm, they demonstrated low loss single mode waveguide of 0.053 dB/cm at 1550 nm. They also demonstrated a refractive index (RI) measurement device based on the evanescent field interaction losses at the surface of the glass^14^.

Fs laser functionalization of glassy material was introduce in 1996 by Davis et al.^15^. Such process uses non-linear effects such as multiphoton absorption or tunnel ionization^16^ to cause permanent changes in the RI. The non-linear dependence on the electric field strength localizes the RI change only at the focal volume which permits the 3D modification into the bulk of the material. The nature of the RI change is highly dependent on the material and writing condition and arises from the sum of many contributions such as color center formation^17^, structural changes in the glass matrix^18^, or thermal effects leading to a change of density^19^, to name a few. There is also a particular regime of thermal accumulation at high repetition rate which gives rise to large out-of-focal RI modification^20^.

Following the research of Lapointe et al., the integrity of the mechanical properties of the protective glass layer by such fs laser modification was studied and it was found that fs writing had negligible impact on the strength of the glass^21^. In the same study, it was shown that the RI change can be increased by one order of magnitude by reducing the number of photons needed (reducing the wavelength) involved in the writing.

In this paper, we first demonstrate a new writing regime without heat accumulation, which leads to highly resolved fine writing points with a positive index change. A positive index change is of particular interest for waveguide writing, while small index change region is critical to write grating with fine periods. This regime is not limited to a single glass, as we demonstrate it in two different glasses. Using this novel writing technique, we demonstrate a volume phase grating operating in the Raman–Nath regime^22^ (VRNG) in front of the smartphone camera to generate an integrated smartphone spectrometer. The key is to produce a weak VRNG that does not significantly alter the traditional function of the camera but generates a spectrum when exposed to bright light illumination. Therefore, we propose to evaluate the possibility of using fs-written volume gratings in those glasses to realize an integrated spectrum analyzer using the smartphone RGB camera.

## Methodology

The fs laser writing was performed using an 8 W Pharos laser system from Light Conversion featuring a 250 fs pulse length. This laser is coupled to an Orpheus OPA to double the frequency from the original 1030 to 515 nm. The fs laser pulse was focused with a 50×, Olympus PLAN 0.65 numerical aperture (NA) microscope objective and the sample was put on a 3-axis writing system controlled by an AEROTECH 3200 controller. The repetition rate of the laser was controlled using a pulse picker to conserve the pulse energy. The polarization of the laser was parallel to the writing direction. The writing speed used was varied from 0.1 to 100 mm/s and the pulse energy from 82 to 825 nJ. Two types of glass were used for the writing, Corning Gorilla glass which is an alkali-aluminosilicate glass used for protection of multimedia screen device and tempered aluminosilicate glass which is a generic add-on removable protective layer from Bodyguardz easily available commercially. To measure the RI change induced, the Ripper system on loan from Photonovainc.com was used. This microscopy interferometric systems measure the relative phase change from the surrounding and, providing knowledge about 2D cross section profile of the inscription, could determine the RI change profile by using the following Eq. (1)^23^.

1 $$ \Delta n = \frac{\Delta \varphi \lambda }{{2\pi h}} $$

where, *Δφ* is the measured phase change, *λ* the wavelength of measurement, which is 633 nm for that system and *h* is the height of the structure. The cross-section was measured with a classical microscopy system with a 60× (0.8 NA) PLAN Olympus objective in the visible.

**Figure 1:**
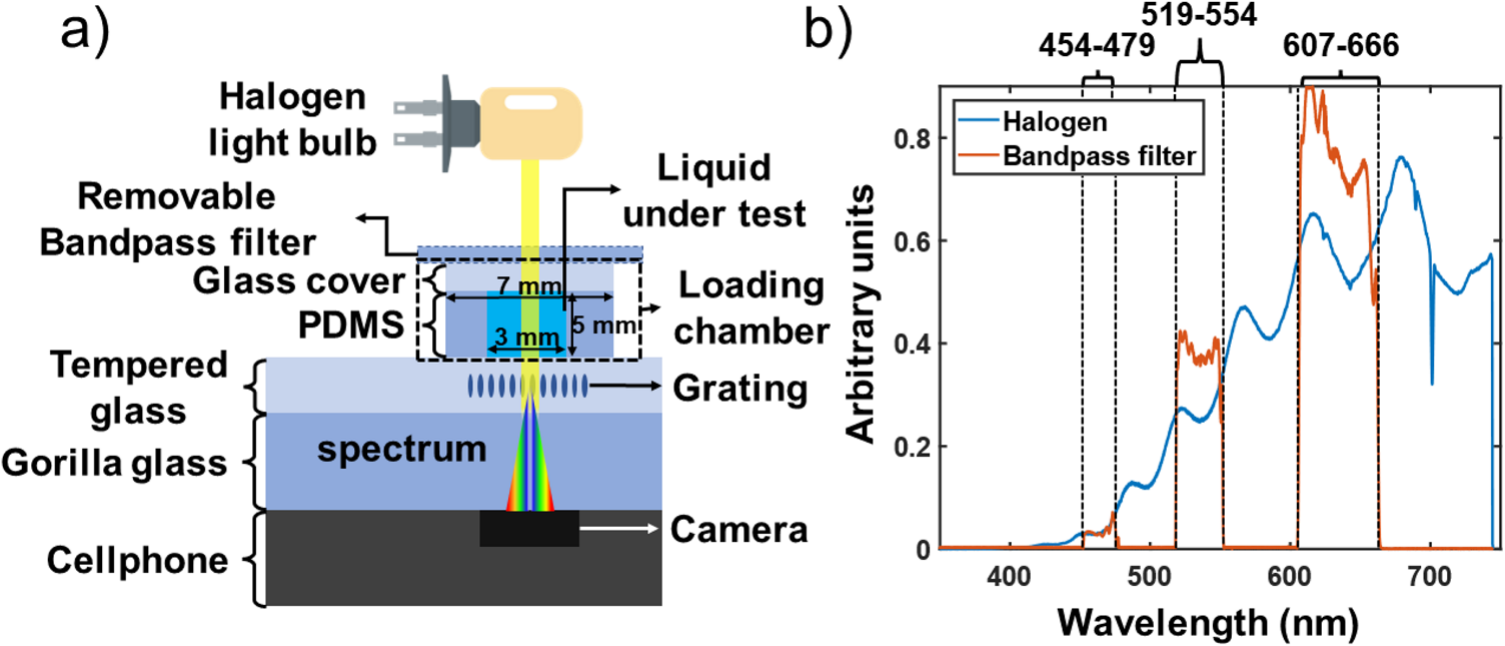
Schematic of principle of the spectrometer on smartphone. Here the grating is written on the removable tempered glass add-on protective layer, but it could also be integrated into the Gorilla glass itself. A loading chamber composed of PDMS can be added on top of the system to measure liquid absorption. In (**b**) we show the spectrum of the halogen lamp without the sample and with the calibration bandpass filters in front of the grating to calibrate the spectrometer.

The cellphone used in this experiment was a Samsung Galaxy S21 FE featuring a 18 MP front camera in front of which the VRNG was placed to acquire the wanted spectrum as represented on Fig. 1a). A loading chamber composed of polymethyl-siloxane (PDMS) of 5 mm thickness and 3 × 3 mm was put in front of the camera as shown on Fig. 1a). A glass slide was put on the top of the chamber to seal it after the liquid is introduce. PDMS was selected since it sticks well to glass creating a sealed cavity. A small Halogen light source of 10 W was set at a distance of 15 cm from the front camera and the spectrum of the light source was characterized with a CCS100 spectrometer from Thorlabs as shown on Fig. 1b). To calibrate the recorded spectra, a 3-band bandpass filter was used instead of the sample. Its transmission spectra with a halogen bulb illumination is shown on Fig. 1b).

## Result and discussion

### Writing regime

**Figure 2:**
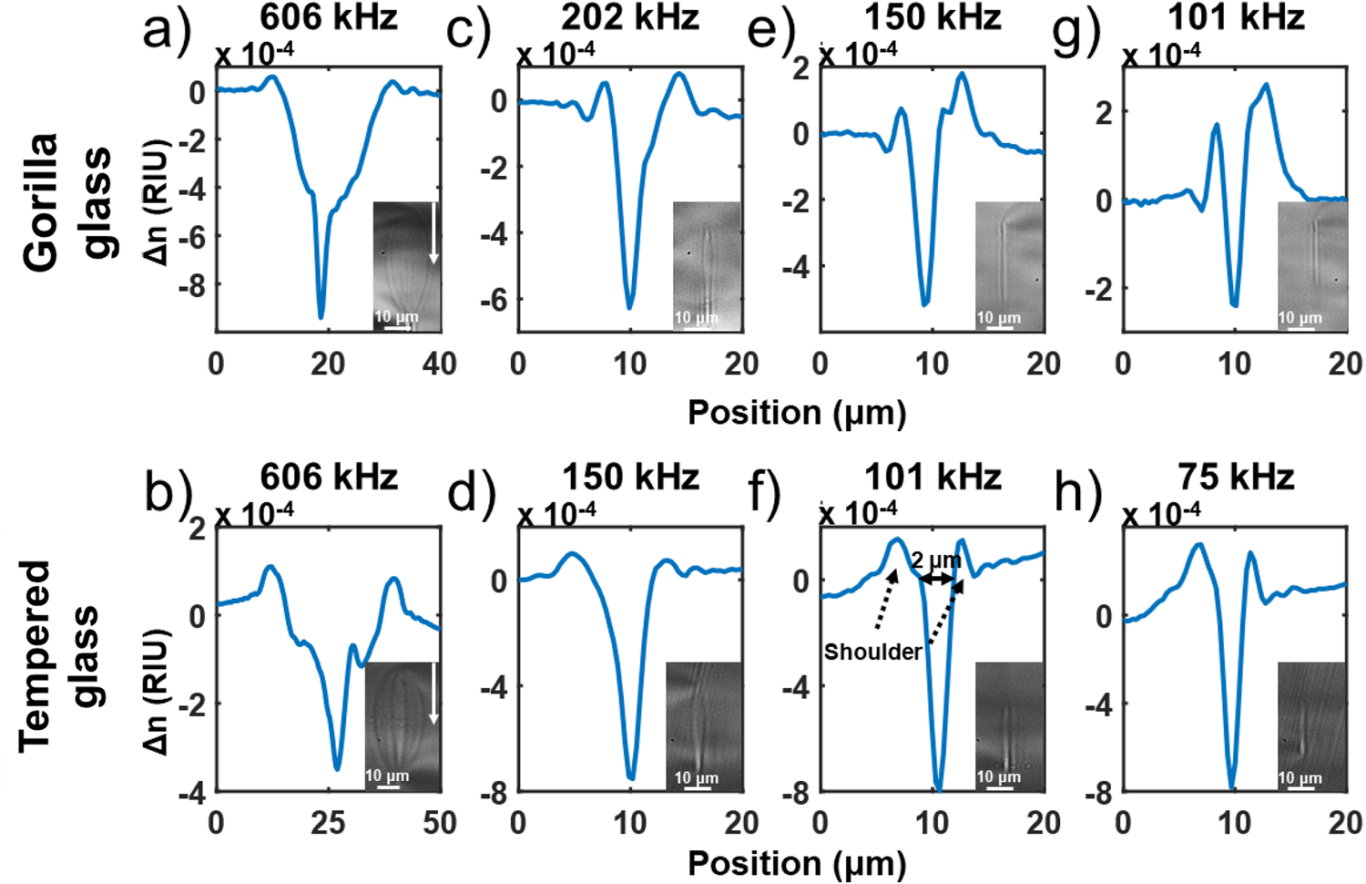
Integrated RI profile tomography and cross-section picture showing the transition and the contribution of thermal accumulation on the RI profile after fs exposure for Gorilla and tempered glass at a writing speed of 50 mm/s. In both cases, the structure is narrower outside of the heat accumulation regime. The white arrows on the cross-section pictures indicate the laser direction. Bigger pictures of the cross-section inset are provided in supplementary material.

In our previous study on fs laser writing into Corning Gorilla Glass^21^, we have shown a thermally driven RI change affecting a large area beyond the focal point, thus generating a 20 by 40 μm multilayer structure. Such a structure is too large if one wants to make a grating of a few microns period. Eaton et al*.*^20^ have simulated that by lowering the repetition rate below 100 kHz in fused silica, thermal accumulation is significantly reduced between each pulse since heat has time to dissipate before the arrival of the next pulse. In order to characterize the impact of such thermal accumulation in our glasses, we have incrementally reduced the repetition rate of our laser with a pulse picker while keeping all the other writing parameters constant (*λ* = 515 nm, *v* = 50 mm/s, *E*_*p*_ = 825 nJ, *NA* 0.65). The results are shown in Fig. 2.

**Figure 3:**
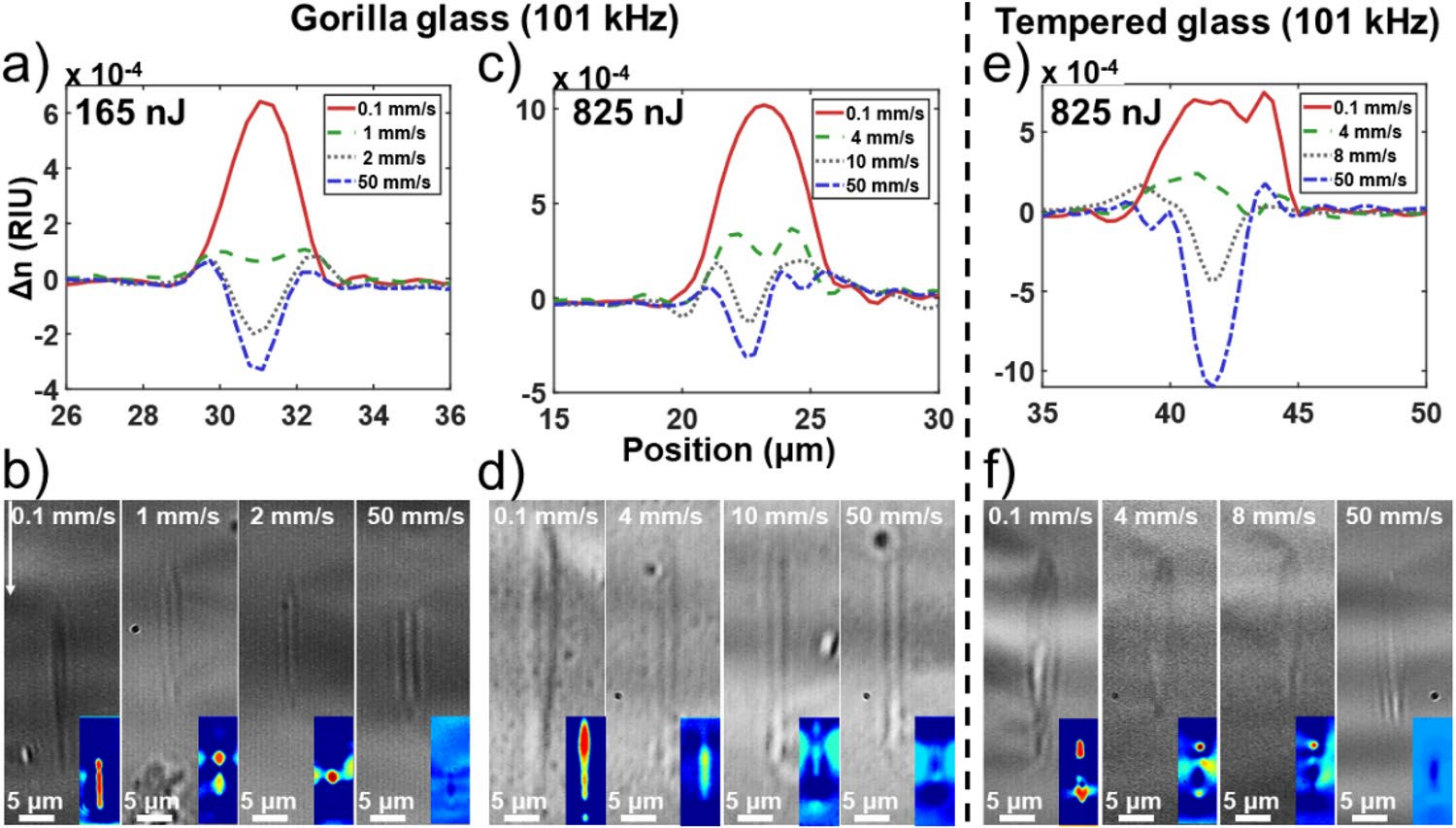
Evolution of the induced integrated RI profile tomography change under fs exposure for different writings speeds at a repetition rate of 101 kHz in Corning Gorilla (**a**–**c**) and tempered aluminosilicate glass (**e**). (**b**,**d**,**f**) are bright-field images of the inscribed cross-sections for different writing speeds, while the colored inset is the output transmission field under white light illumination showing either single-mode or multi-mode propagation or no propagation at all. The illumination scale goes from blue (low light) to red (high brightness) relative to the surrounding.

As one can observe in Fig. 2, when outside of the heat accumulation regime (e, f), narrower and elongated structures are written, with significantly higher eccentricity. This transition to this regime occurs gradually around 150 kHz for Gorilla glass (Fig. 2e) and 101 kHz for tempered glass (Fig. 2f), which suggests that Gorilla glass has a slightly higher heat diffusion rate than the tempered glass. Below such frequencies, the RI profile is composed of a narrow peak of negative index (~ 2 μm) with small positive shoulders. Such a narrow structure could come from filamentation since the peak power for a 825 nJ pulse is P_p_ = 3300 kW, which is above the self-focusing power threshold (P_th_ = 550 kW) defined by Eq. (2)^24^.

2 $$ P_{th} = \frac{{\alpha \lambda^{2} }}{{8\pi n_{0} n_{2} }} $$

where α is a constant related for the spatial beam distribution for a Gaussian beam which is equal to 3.77, *λ* the center wavelength of the pulse, *n*_*0*_ the linear RI which is 1.523 and *n*_*2*_ the non-linear RI coefficient which is 4.77 × 10^–20^ m^2^/W for aluminosilicate glass^25^. We then attempted some optimization of this regime by varying the speed from 0.1 to 50 mm/s and at a constant pulse energy between 165 and 825 nJ at a repetition frequency of 101 kHz for both glasses. The results are shown in Fig. 3.

One can observe in the 3 cases reported in Fig. 3 that there is a transition from a negative to positive RI when increasing the fluence, either through writing speed reduction or pulse energy increase, for the two different types of glass. The negative RI change at low fluence is believed to be attributed to the quenching of the glass at a lower fictive temperature than the glass thermal history thus reducing the local density^19^. The positive RI change could result from accumulation of color centers^17^ or from structural restructuration favoring the 3–4 Si–O ring members at the expanse of the 5–6 ones^18^. Note that the guidance observed in Fig. 3b,d,f) in negative index waveguides is not centered on the main index change and is likely due to positive index artifact around the center. Such positive index change is of high interest for waveguide writing. This positive index writing regime does not appear to be specific to one glass, but the transition threshold speed and frequency threshold are dependant on the material. Consequently, the transition appears to be fluence driven with the fluence (*F*) definition express at Eq. (3)^26^.

3 $$ F = \left( {\frac{{R_{r} \omega_{0} }}{v}} \right)\frac{{E_{1p} }}{{\pi \omega_{0}^{2} }} $$

where *v* is the writing speed, *R*_*r*_ the repetition rate, *E*_*1p*_ the pulse energy and *ω*_*0*_ the waist of the beam for 0.65 NA microscope objective. From our results, positive index change occurs for a fluence above 8.7 × 10^6^ J/m^2^ for Corning Gorilla glass and 1.4 × 10^7^ J/m^2^ for the tempered aluminosilicate glass. On the cross-section view at Fig. 3b,d,f), it can be observed that an increase in fluence elongates the RI change region (increases the eccentricity of the ellipsoid). This elongated feature is likely due to filamentation into the glass. Lower pulse energy for the same fluence generates finer structure. One must be careful when using this writing regime for waveguides, as such waveguides may become multi-mode in the vertical direction.

### VRNG writing parameters

**Figure 4:**
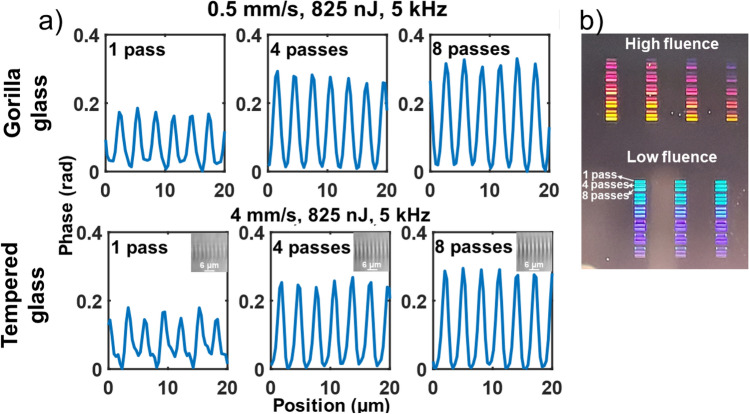
(**a**) Phase changes for the best recipes from the parameter grid presented in (**b**), which shows diffraction patterns in transmission for the 3 μm pitch VRNGs written at high and low fluence in Gorilla and tempered glass for various number of passes. The depth of the inscription increases with the number of passes which explains the increase in phase change. The scan parameters used were from 0.1 to 4 mm/s, 1 to 8 passes, 125 to 865 nJ of pulse energy and repetition rates set with a pulse picker of 5 kHz and 80 kHz. Bigger pictures of the cross-section inset are provided into supplementary material.

To inscribe VRNGs, a high RI change in a very thin elongated region is required. The writing regime proposed in the last section is ideal for this. To optimize the writing of VRNG structures, we compare two writing regimes: one at low pulse energy with high fluence and the other at high pulse energy with low fluence. To this end, a grid of small VRNGs composed of 30 lines with a 3 μm pitch have been written with different fluences (from 6.5 to 104 × 10^6^ J/m^2^ and from 1.3 to 34 × 10^6^ J/m^2^) and different number of passes (1 to 8 passes). A 3 μm pitch was selected to ensure the highest line density without overlap between inscription lines. These results are displayed in Fig. 4. For a VRNG, the total phase change ($\Delta \phi \propto\Delta n\cdot h$) is more relevant than the RI change since the efficiency of the grating is determined by the phase change induced. For instance, a π rad phase change minimises power in the 0^th^ order and thus maximises power into diffraction orders.

As shown on Fig. 4, in both cases the best recipe was found at the higher pulse energy and lower fluence cases which produces a phase change of 0.3 rad. Multiple passes increases the cross-section length by a factor 1.67 yielding the same factor of increase in phase change. Both glasses produce similar maximum phase change but the writing speed in tempered glass is 8 times faster, therefore this glass has been selected for the application demonstration.

### Cellphone integrated spectrometer

**Figure 5:**
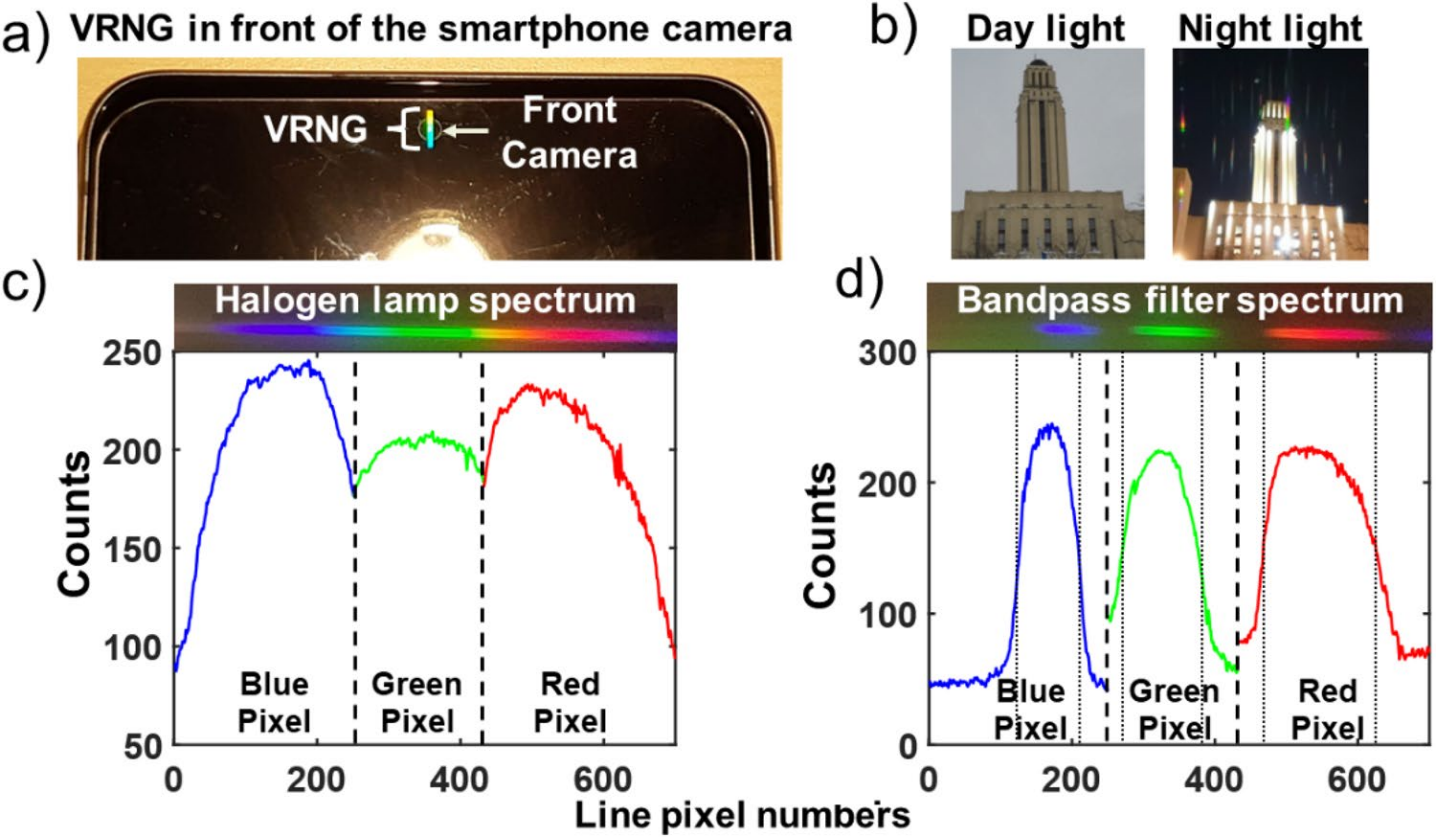
(**a**) VRNG inscribed into tempered glass and placed in front of the smartphone camera. (**b**) The grating does not affect the day-light picture quality taken with the camera if no bright sources are present, but the diffraction spectra does appear if a bright light is brought close to it or in a low light environment. In (**c**), we show the 2nd diffraction order spectrum recorded by the camera generated by the VRNG from the halogen lamp and with the calibration filter in front of it (**d**). Each spectrum recorded is averaged over 3-pixel line to reduce the noise.

A 3 by 0.5 mm VRNG was inscribed 100 μm under the surface of the tempered glass and placed in front of the smartphone camera as described in Fig. 1a and shown on Fig. 5a. In normal day light illumination, the grating has no effect on the image taken by the camera as long as there is no bright light point source within the image (for instance, the sun). However, in a low light environment, parasitic spectra will appear in pictures for every light source as shown on Fig. 5b. Hence, the VRNG can be kept on the camera in many situations without impacting the main functionality of the device. To demonstrate its use as a spectrometer, we first calibrated the spectrometer using a bandpass filter as described in Fig. 1b. The calibration filter was placed in front of the glass and illuminated by a 10 W halogen lamp as shown on Fig. 1a. The grating caused a diffracted spectrum to appear on the pixel grid of the camera. The best results were using the 2nd diffraction order. Along this spatially distributed colored pattern, only the red, green or blue (RGB) pixels yielding the maximum signals were used to sample the spectrum. Therefore, the spectrum was divided into 3 bands: red, green and blue. This strategy was used to counteract the possible crosstalk between diffraction orders such as the 2nd (used) and the 3rd (parasitic) orders. The detection band and isolation are shown in supplementary material.

One can observe the match between the recorded spectra and the RGB fragmentation detection scheme presented at Fig. 5c and d for the halogen lamp and the calibration filters. The edges at half maximum of each band produced by the calibration filters were used to set 6 wavelength points for calibration. Then a 2nd order polynomial fit was used to find the calibration function as shown in Eq. (4).

4 $$ \lambda \, = \, 0.000012{\text{ m}}^{2} + \, 0.4{\text{ m }} + \, 400 $$

where *m* is the pixel number. Despite the theoretical sinusoidal behavior of the diffraction, the calibration function was found to be quasi-linear with a detector resolution of 0.4 nm/pixel. This linear dispersion comes from the use of the VRNG in the small angle approximation regime. The optical resolution of the detector was measured using a Helium Neon laser line centered at 632.8 nm and was found to cover 9 pixels which represent 3 nm at full width at half maximum. Using a finer writing pitch would increase the dispersion of the grating and thus the resolution of the spectrometer. However, a fine pitch—if it can be resolved through the writing—brings a higher diffraction angle which may result in the spectrum image outside of the field-of-view of the camera. The 3-µm pitch grating used produces a full 2nd order spectrum that extends up to 90% of the field of view of the camera. The diffraction power efficiency at the 2nd order was measured to be 0.3% of the incident light at 632.8 nm. The form factor of the grating is well suited for utilizing the phase mask writing scheme which has the advantage of not only increasing the resolution but most importantly to drastically reduce the production time to allow mass production.

### Spectrometer testing example

**Figure 6:**
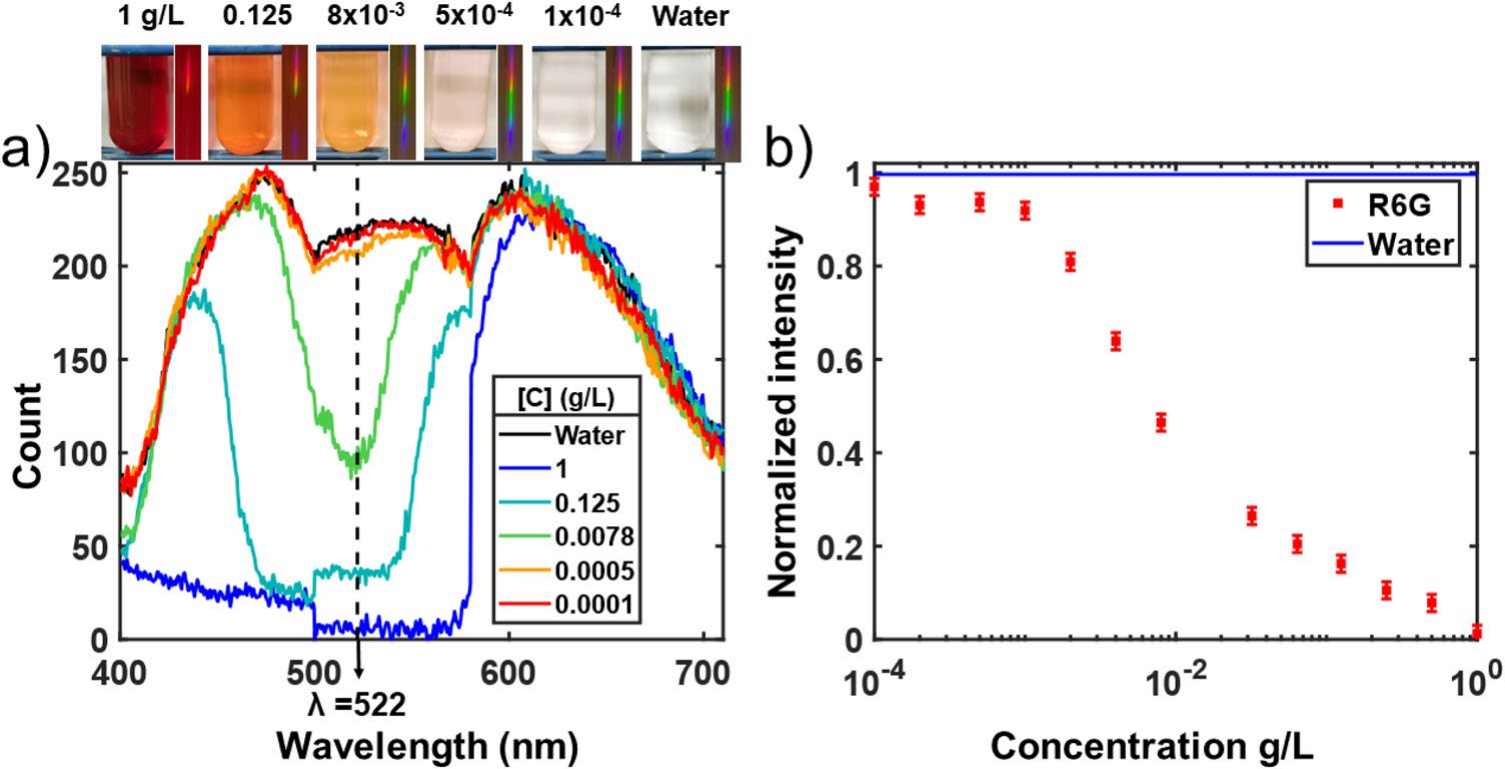
(**a**) Raw absorption spectrum data produced by different concentrations of rhodamine 6G in water. The recorded spectrum from each concentration is shown in the inset. Raw data is displayed to show physical device performance and limitations. (**b**) The intensity at λ = 522 nm for all the concentrations used normalized to the water spectrum. The signal variation at 500 nm comes from different gains parameters between the RGB bands, which is performed internally in the firmware of the camera.

As a proof of concept using this calibrated spectrometer, the absorption spectrum produced by different concentration of rhodamine 6G in water put into the loading chamber presented in Fig. 1a is shown in Fig. 6a. The initial concentration was taken at 1 g/L.

The spectra presented in Fig. 6a are the average result of 3 lines of pixels to reduce the noise. The remaining noise was quantified by processing 10 pictures of the water spectrum and was determined to be ± 0.02 units at 2σ (95% confidence interval) as shown in the error bar of the water point data in Fig. 6b. The upper limits of the concentration error introduced by that noise can be evaluated by linear interpolation of the lowest variation of the curved profile in Fig. 6b occurring near the concentration detection limits around 0.001 g/L. Doing so, the error is found to be of 0.4 mg/L. One can observe in Fig. 6a that the 0.1 mg/L spectrum overlaps with the water spectrum, but a small difference is observed for the 0.5 mg/L spectrum. The absorption peak is found near λ = 522 nm which correspond to the expected peaks of rhodamine 6G in water which is slightly shifted from 530 nm in ethanol^27^ as reported in^28^. Therefore, the limit of detection of rhodamine 6G is found at 0.5 ± 0.4 mg/L for a 5 mm thick loading chamber. A better control over the cameras acquisition parameters could offer an opportunity to stretch the detection limits, standardize the spectrum across the RGB detectors and linearize the detection.

Rhodamine 6G was used in this study since it is a well know dye with a strong absorption band in the visible. Not all analytes of interest have as high a coefficient of absorption which lead to higher limits of detection. This could be problematic for certain applications like water contamination evaluation where concentrations are in the order of the mg/L. To mitigate this difference in efficiency, the thickness of the loading chamber can be increased which will have an exponential impact on the detection limits. However, indirect detection systems could be also evaluated by measuring the impact on an efficient absorber—such as rhodamine—produced by the presence of an analyte. Thereby, a selective Cu^2+^ colorimetric sensor was demonstrated capable of detection concentration in the μg order using rhodamine derivative^29^ as well as for lead^30,31^ and cadmium^30^ paving the way to useful field spectrometer.

## Conclusion

Demonstration of an integrated spectrometer based on the inscription of a small weak VRNG in front of a smartphone camera was reported using a removable protective aluminosilicate tempered glass cover layer. Such VRNGs were also demonstrated in Corning Gorilla glass which opens the possibility of spectrometer integration directly into the smartphone glass layer (no removable device). In an effort to produce optimal RI change by fs writing for VRNGs, a new writing regime without thermal accumulation was characterized. The thermal accumulation upper-bound thresholds for both glasses were measured at a repetition rate of 150 kHz and 101 kHz for Corning Gorilla glass and aluminosilicate tempered glass, respectively. Below such frequencies, a transition from negative (low fluence) to positive (high fluence) RI change was observed. The fluence required to reach positive index change regime is 8.7 × 10^6^ J/m^2^ and 1.4 × 10^7^ J/m^2^ for each of the respective glasses. Those regimes were investigated for VRNG production where a high phase change in a narrow region is preferred. Such optimization showed that a low fluence and high pulse energy, coupled with a multiple pass strategy, is preferable. Weak VRNGs with phase change of 0.2–0.4 rad were incorporated in front of a smartphone camera. It was shown that such integration does not perturb the camera’s main function in high luminosity operation if no bright sources are present. The RGB camera was used as 3-wave-band spectrometer yielding 3 nm optical resolution and 0.4 nm/pixel detector resolution. The spectrometer detection limit was measured using various concentration of rhodamine 6G in water and was found to be of 0.5 mg/L ± 0.4 mg/L. We can therefore conclude that fs-inscribed structures can be integrated—permanently or in a removable fashion—into the smartphone to incorporate a spectrometer functionality which can be used to detect and quantify the presence of analytes. It could be interesting to evaluate the trade-off between the use of a 2nd and a 1st order grating since the latter one could be theoretically more efficient depending on the overlap between the writing line. This demonstration paves the way for absorption spectroscopy on smartphones and further the integration of photonics.

### Supplementary Information

Supplementary Figures.

## Data Availability

The datasets generated during and/or analysed during the current study are available from the corresponding author on reasonable request.
