# Supplementary material for: Fs laser written volume Raman–Nath grating for integrated spectrometer on smartphone
Source: Sci Rep. 2023 Aug 22;13:13717. doi: 10.1038/s41598-023-40909-9 (PMC10444826; doi:10.1038/s41598-023-40909-9)
Supplement: Supplementary file 1 — Supplementary Figures. [file 41598_2023_40909_MOESM1_ESM.pdf]

Supplementary information

Fs laser written volume Raman-Nath grating for integrated spectrometer on smartphone

JEAN-SÉBASTIEN BOISVERT[1*], SÉBASTIEN LORANGER[2] AND RAMAN KASHYAP[2,3]

[1]*Department of Engineering Physics, Ecole Polytechnique Montréal, 2900 Édouard-Montpetit, (QC), Montréal, H3T 1J4, Canada*
[2]*Department of Electrical Engineering, PolyGrames, Ecole Polytechnique Montréal, 2900 Édouard-Montpetit, Qc, Montréal, H3T*
**jean-sebastien-2.boisvert@polymtl.ca*

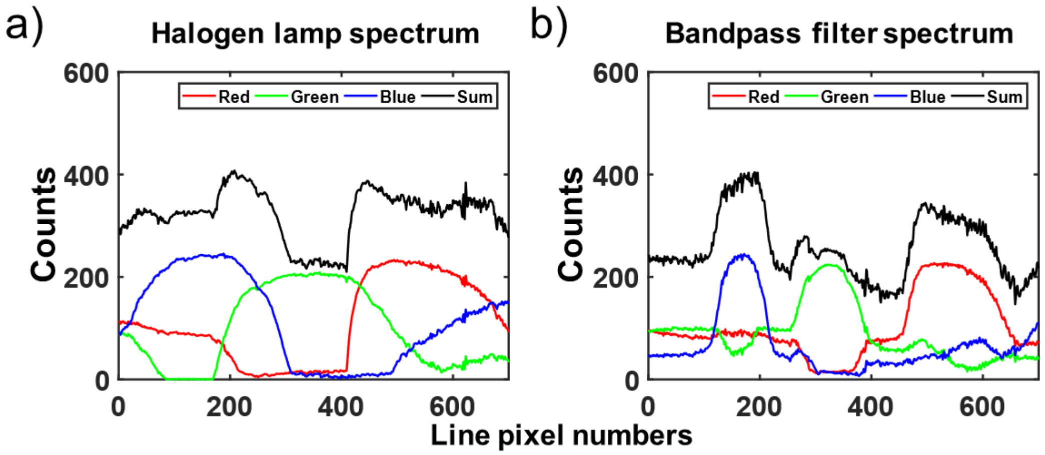

Supplementary Figure 1 RGB components and their total summation for a) the halogen lamp and b) the bandpass filter. The poor isolation of each component of the RGB detector distorts the spectrum.

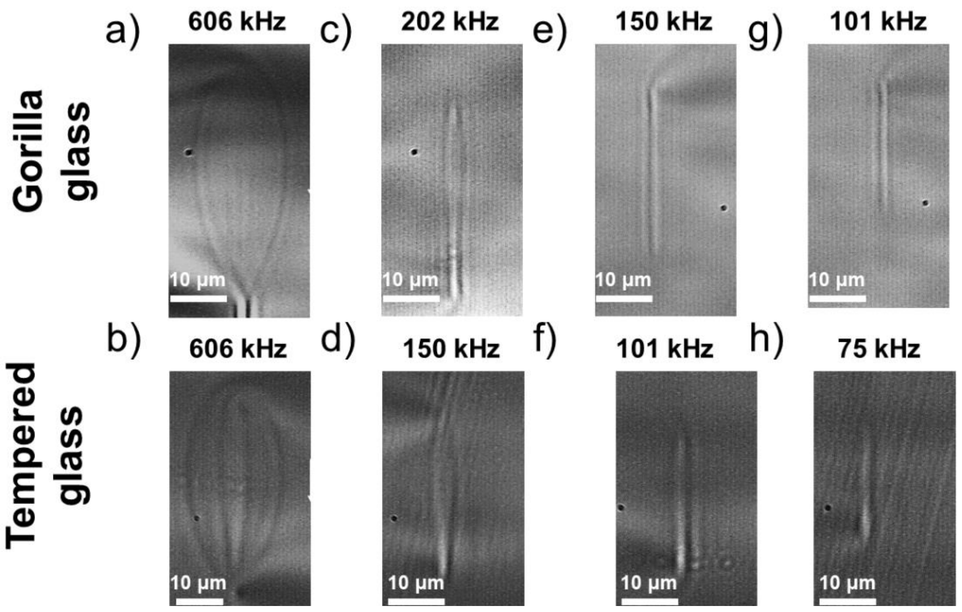

Supplementary Figure 2 Zoom on the inlet picture of figure 2 of the main manuscript showing the cross-section of the inscriptions.

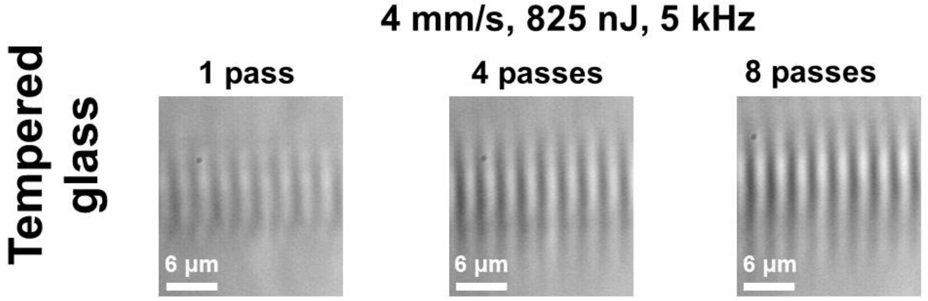

Supplementary Figure 3 :Zoom on the inlet picture of figure 3 of the main manuscript showing the cross-section of gratings.
